# SILVA in 2026: a global core biodata resource for rRNA within the DSMZ digital diversity

**DOI:** 10.1093/nar/gkaf1247

**Published:** 2025-11-18

**Authors:** Maria Chuvochina, Jan Gerken, Martinique Frentrup, Yeliz Sandikci, Robin Goldmann, Heike M Freese, Markus Göker, Johannes Sikorski, Pablo Yarza, Christian Quast, Jörg Peplies, Frank Oliver Glöckner, Lorenz Christian Reimer

**Affiliations:** Leibniz Institute DSMZ-German Collection of Microorganisms and Cell Cultures, D-38124, Braunschweig, Germany; Leibniz Institute DSMZ-German Collection of Microorganisms and Cell Cultures, D-38124, Braunschweig, Germany; Leibniz Institute DSMZ-German Collection of Microorganisms and Cell Cultures, D-38124, Braunschweig, Germany; Leibniz Institute DSMZ-German Collection of Microorganisms and Cell Cultures, D-38124, Braunschweig, Germany; Leibniz Institute DSMZ-German Collection of Microorganisms and Cell Cultures, D-38124, Braunschweig, Germany; Leibniz Institute DSMZ-German Collection of Microorganisms and Cell Cultures, D-38124, Braunschweig, Germany; Leibniz Institute DSMZ-German Collection of Microorganisms and Cell Cultures, D-38124, Braunschweig, Germany; Leibniz Institute DSMZ-German Collection of Microorganisms and Cell Cultures, D-38124, Braunschweig, Germany; Bioinfile SL, Alicante, 03540, Spain; Ribocon GmbH, D-28359, Bremen, Germany; Ribocon GmbH, D-28359, Bremen, Germany; MARUM—Center for Marine Environmental Sciences, University of Bremen, D-28359, Bremen, Germany; AWI—Alfred Wegener Institute, Helmholtz Centre for Polar and Marine Research, D-27570, Bremerhaven, Germany; Leibniz Institute DSMZ-German Collection of Microorganisms and Cell Cultures, D-38124, Braunschweig, Germany

## Abstract

Since 2007, the SILVA database (https://www.arb-silva.de/) has served as a comprehensive resource providing quality-checked, aligned, and classified ribosomal RNA sequences for the scientific community worldwide. The database provides manually curated taxonomic classifications for the three domains of life, standardized reference datasets, and tools for classifying microbial diversity. SILVA’s impact has been recognized in its designation as ELIXIR Core Data Resource and Global Core Biodata Resource. The integration of SILVA into the DSMZ Digital Diversity consortium (D3; https://hub.dsmz.de) in 2023 marked a significant update, ensuring the long-term sustainability and continued development of the resource. Apart from moving to a new hosting institution, this integration facilitates data interoperability and standardization among SILVA, LPSN, StrainInfo, and other D3 databases. New developments this year include a redesigned website consistent with the DSMZ corporate identity, DOI assignments for all SILVA releases to enhance data citation and support the FAIR data principles, and the provisioning of QIIME2, DADA2, and KRAKEN2 classifiers. Additionally, the EukMap application has been redeveloped as a curation and visualization tool called TaxMap. In anticipation of the next data release, we outline recent developments and the current state of the database, its relation with other D3 resources, and its future directions.

## Graphical Abstract

**Graphical Abstract:**
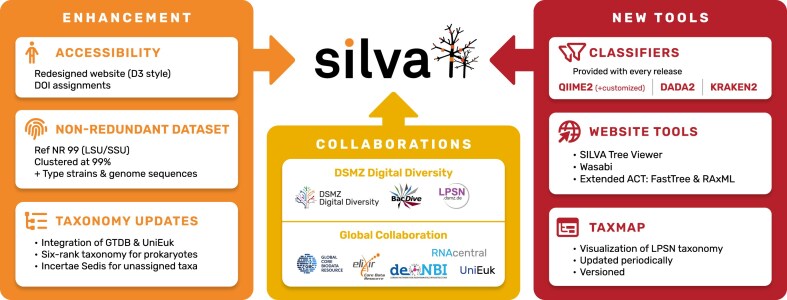

## Introduction

SILVA is a comprehensive resource that provides quality-checked, aligned, and taxonomically classified ribosomal RNA (rRNA) gene sequences across all domains of life. It is also the sole resource providing rRNA gene-based classifications and analyses tools based on both small (SSU) and large (LSU) ribosomal subunits. One of the important features of SILVA is the manual curation of its taxonomies from genus to domain, which integrates knowledge from widely used resources on nomenclature and classification. This includes List of Prokaryotic names with Standing in Nomenclature (LPSN; [1]), Genome Taxonomy Database (GTDB; [2]), NCBI Taxonomy [3], and UniEuk [4]. The development of SILVA has been documented in three key publications in the Nucleic Acid Research Database Issue [5–7]. These publications have been cited over 41 000 times in total, including ~10 000 citations since 2024 (Google Scholar, August 2025). Receiving visits from users from 193 countries (since 2024; Matomo, August 2025), SILVA serves as one of the most widely used global resources in microbial taxonomy and as a central repository of quality-screened rRNA sequences. Apart from regular applications such as taxonomic profiling in metabarcoding surveys (e.g. [8]), SILVA serves as a reference database in taxonomic classifiers [9–11] and has been used in the generation of new classification resources [12, 13]. SILVA is one of the Expert Databases integrated within RNAcentral, a central resource for the RNA community that aggregates data from specialized noncoding RNA (ncRNA) resources and provides unified access to ncRNA sequences from a wide range of species [14]. SILVA also serves as one of the external resources in both ENA’s cross-reference system and NCBI’s LinkOut service, providing direct links from their sequence records to corresponding SILVA entries [3, 15]. Recognition of the high scientific value and services provided by SILVA led to its designation as an ELIXIR European life science infrastructure in 2018 [16] and a Core Data Resource by the Global Biodata Coalition in 2023 [17].

Recently, SILVA has been integrated into the Digital Diversity consortium (https://hub.dsmz.de) at the Leibniz Institute DSMZ—German Collection of Microorganisms and Cell Cultures to ensure its further development and integration with other well-known DSMZ Digital Diversity databases such as LPSN [1], Bac*Dive* [18], and StrainInfo [19].

Here, we present and discuss major updates of SILVA, including its integration into the DSMZ Digital Diversity consortium, the redesigned website, the addition of classifiers to the SILVA pipeline, and the increased adherence to the FAIR data principles. In addition, we describe methodological and curation updates since the last release, and the introduction of TaxMap—a new taxonomy visualization and curation aid tool. We expect the new release to be available online by the end of 2025 due to the significant increase in the amount of sequence data requiring curation. Finally, we discuss the future development of the database following its integration into DSMZ Digital Diversity.

### Integration into the DSMZ digital diversity

Starting in 2023, SILVA officially joined the newly established DSMZ Digital Diversity consortium (D3; https://hub.dsmz.de), which currently encompasses the development of nine life science databases, four of which are Global Core Biodata Resources at the Leibniz Institute DSMZ—German Collection of Microorganisms and Cell Cultures in Germany. SILVA’s move to the Leibniz Institute DSMZ, one of the world’s largest collections of microorganisms and cell cultures, represents a major milestone in securing funding for continuous development and long-term sustainability as a Core Data Resource in life sciences. Therefore, this transition enables us to strategically plan for the modernization of the database and its associated services. Furthermore, integrating SILVA into the D3 Hub, a central platform that brings together scientific databases operated at DSMZ [18], improves database interoperability and cross-linking with other D3 resources. The central goal is to provide access to integrated data covering a wide range of biological entities, enabling new scientific discoveries and insights. SILVA has already been integrated with LPSN [1] and Bac*Dive* [18], two prominent D3 resources. Linking SILVA to nomenclatural information from LPSN allows users to select relevant sequences based on their association with taxa that have validly published names or pro-valid *Candidatus* taxon names for inclusion in phylogenetic analyses. Meanwhile, integration with Bac*Dive* enables users to collect comprehensive metadata along with rRNA gene sequences, which could, for example, help gain insights into the evolution of specific features or their presence/absence across particular lineages. To further expand these existing integrations, we plan to connect SILVA’s taxonomic and rRNA sequence data with enzymatic data from BRENDA [20], which would facilitate exploration of metabolic patterns across different organisms and contribute to the understanding of evolutionary relationships of enzymatic functions across taxa. Another example of integration involves the recently re-established StrainInfo [19], which provides strain deposition history and resolves strain identifiers from different culture collections. This would allow users to easily navigate connections between sequences derived from type strains and those representing the same species according to the original submitters, helping to identify potential misclassifications and improve taxonomic accuracy by comparing its placement in SILVA. To achieve these goals, a federated search has been developed across all D3 databases (https://hub.dsmz.de/#/search), including sequences from SILVA datasets. We are committed to further improvements in data and term standardization, as well as facilitating data retrieval through API development (see Future Directions). In summary, SILVA’s integration into the DSMZ Digital Diversity will significantly improve database interoperability and establish a robust foundation for serving the global scientific community.

### Workflow and content changes

**Figure 1.**
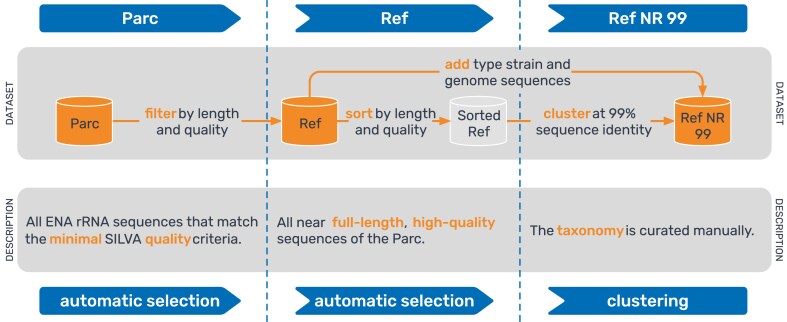
Workflow for processing and organizing rRNA sequence data into three SILVA datasets, Parc, Ref and Ref NR 99, according to the sequence quality criteria.

Since our last update on SILVA [7], we have implemented numerous methodological and curation changes to improve the resource. Key changes include the introduction of nonredundant reference dataset (Ref NR 99) for SSU and later for LSU, starting from release 115, which clusters sequences at 99% identity threshold to remove highly identical sequences from the reference dataset (Ref) and guide trees, resulting in improved computational efficiency and cleaner phylogenetic trees. Sequences derived from type strains of cultured species are subsequently re-added after clustering, as many serve as nomenclatural types for higher taxa and facilitate name application within the tree. Up to release 138.2, sequences from the ENA and NCBI genome list were also re-added after clustering (Fig. 1). The dataset growth (Supplementary Fig. S1A–F) also prompted the transition to calculating guide trees based only on the Ref NR 99 datasets (starting from release 115 for SSU and from release 138.1 for LSU), whose taxonomy is manually curated and subsequently propagated to the Ref NR sequences that fall within the same 99% identity clusters. In addition, automatic sequence classification has been added for the full dataset (Parc) based on the SILVA taxonomy derived from the Ref NR 99 dataset (starting from release 119). In summary, the Ref NR 99 datasets contain a representative subset of sequences that maintains phylogenetic diversity while being computationally feasible for tree inference with the growing dataset.

To optimize the performance and ensure continuity between releases, we changed the clustering approach for the Ref NR 99 dataset from UCLUST to VSEARCH [21] in SILVA release 138. VSEARCH is more accurate compared to UCLUST and allows clustering by similarity using user-defined order [21]. The clustering process is performed using 99% identity threshold according to a custom sequence order based first on presence in the last release’s Ref NR 99 and second on combination of sequence length (weighted 2-fold) and quality. This approach preserves representative sequences from the previous release while newly added sequences are clustered using length-dominated pre-sorting, ensuring that the longest sequences are more likely to be selected as new representatives for subsequent placement into the tree. Furthermore, the transition to VSEARCH, an open-source and freely available tool, contributes to improving the transparency and reproducibility of the SILVA pipeline and supports the FAIR data principles.

Since 2023, we use the de.NBI cloud [22], operated by the German Network for Bioinformatics Infrastructure (de.NBI), to process jobs submitted via the SILVA website. This change ensures timely processing of all jobs, reduces the computational load on SILVA’s infrastructure, and improves failover capacity. As a partner of the de.NBI project, we believe that this solution also enables reliable and scalable computational resources for the entire life science community and promotes efficient use of data in research.

In terms of metadata and links to external resources, we added links to Bac*Dive*, LPSN and the DSMZ catalog that can be accessed from the individual sequence pages under General information. In addition to ENA and other taxonomies, we have added the Genome Taxonomy Database (GTDB) [2] as a new alternative taxonomic source to the metadata fields in our ARB export files (tax_gtdb) and website. GTDB provides a rank-standardized and phylogenetically consistent classification for bacterial and archaeal genomes and has been included since SILVA release 138 for genome-derived sequences. When taxonomic mapping is unavailable due to lack of genomic representation, sequences are assigned as “unclassified.” In addition, information about genome-derived sequences in SILVA is now imported from NCBI and tagged n[G] in the sequence metadata, replacing the previous e[G] tag that was used to indicate the former source database, EMBL. To provide taxonomic category information for names in SILVA, we added mapping files and categories for SSU and LSU taxonomies (from release 119). With respect to SSU resources, the All-Species Living Tree Project (LTP) releases were based on SILVA data and hosted by SILVA from 2008 to 2020. Since LTP_2020, the LTP team maintains its own website for newer releases [23], while previous LTP releases remain available in the SILVA archive.

Regarding the SILVA tools and website, the SILVA Tree viewer [24] was integrated in 2017. This web application enables users to visualize and navigate the large phylogenetic trees provided by SILVA. Users can query SILVA’s guide trees by accession numbers or taxon names and visualize associated metadata including isolation source, sequence length, and strain identifiers. Another visualization tool, Wasabi [25], was added to the website in 2018 to support visualization and assessment of alignments generated through the SILVA ACT (Alignment, Classification, and Tree Service) tool. The same year, the search and classify options in the ACT tool were expanded to include reconstruction of maximum likelihood trees based on user and/or SILVA aligned sequences using FastTree [26] or RAxML [27] software. Finally, as of release 138, the terms of use of SILVA database, its taxonomy, and all downloadable files were updated to Creative Commons Attribution 4.0 (CC-BY 4.0). Note that earlier SILVA releases are not affected by the updated license, and the earlier license information is provided for each release before 138 in the download directory.

### Curation and taxonomy changes

SILVA remains the sole taxonomic database which provides rRNA-based classification for the three domains of life based on both LSU and SSU ribosomal subunits. Starting from release 138, we integrated additional taxonomic resources for manual curation to ensure that the latest developments are reflected in SILVA taxonomy. We adopted the genome-based taxonomy for prokaryotes [13, 28] and UniEuk [4] for protists, which together enable more accurate taxonomic classifications. Where possible, we reused existing GTDB placeholder names for prokaryotic taxa lacking Latin names and/or those delineated based on rank-normalization approach [13]. The adoption of such names aims to promote consistency between taxonomic resources and facilitate comparisons between 16S and genome-based classifications. Other established resources for prokaryotic taxonomy and nomenclature such as Bergey’s Manual of Systematics of Archaea and Bacteria and LPSN database have been adopted to assist with the curation and ensure the appropriate name application (e.g. LPSN correct names). In recent years, prokaryotic nomenclature went through some major revisions including recognition of the phylum as an official taxonomic category under the International Code of Nomenclature of Prokaryotes (ICNP) [29], recognition of the categories of domain and kingdom under the ICNP [30], solving problems with class names by making Rule 8 of the ICNP nonretroactive regarding the formation of such names [31, 32], establishment of Candidatus Lists with continuous cataloging of such taxa in International Journal of Systematic and Evolutionary Microbiology [33], and establishment of a new nomenclature code, SeqCode, based on genome sequences as type material [34]. Starting from release 138.2, SILVA adopted 43 validly published phylum names, which occur in both SSU and LSU taxonomies, under the ICNP for *Bacteria* and *Archaea*. In terms of classification changes, since the majority of prokaryotic taxa are classified at the principal taxonomic categories (e.g. see GTDB or LPSN taxonomies) and in alignment with GTDB, we established a six-category and therefore six-rank (genus, family, order, class, phylum, and domain) taxonomy for *Bacteria* and *Archaea* in SILVA. In the same 138.2 release, we introduced *Incertae sedis* as a placeholder name for unassigned sequences at different ranks and taxa known only from environmental sequences. As an example, if a group with a Latin or placeholder name is missing a named parent, *Incertae sedis* is applied as a parent name. The application of this new naming scheme resulted in creation of 2726 *Incertae sedis* taxa including 1524 genera, 776 families, 279 orders, 112 classes, and 12 phyla (SSU: 1146 genera, 572 families, 194 orders, 75 classes, 8 phyla; LSU: 378 genera, 204 families, 85 orders, 37 classes, 4 phyla). Subsequently, we discontinued the use of the term “uncultured” for taxa known only from environment sequences since the application of this term is ambiguous and not all of the sequences representing such taxa might be from uncultured organisms, making this term misleading.

## Recent developments

### Integration into DSMZ brand

**Figure 2.**
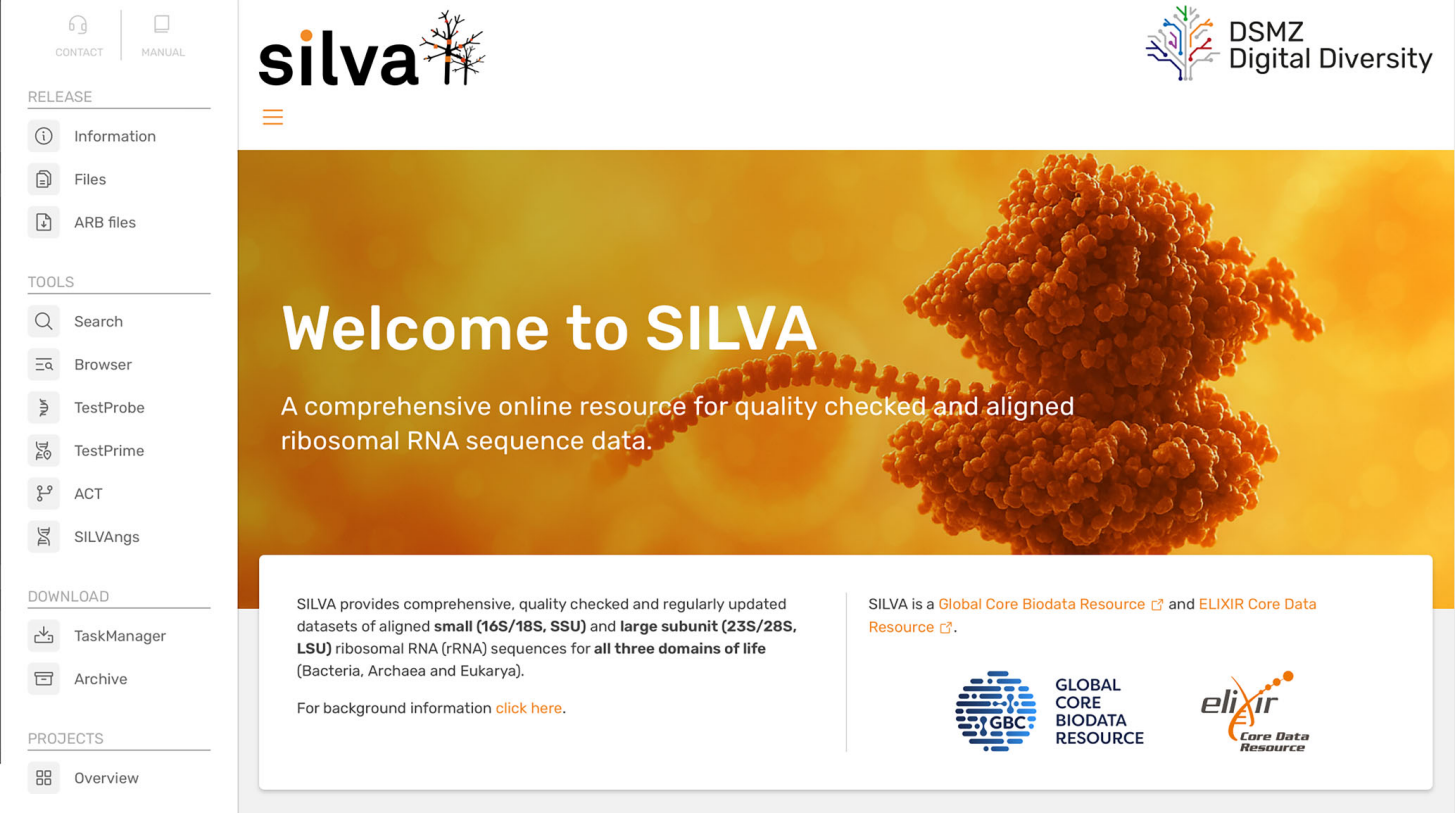
SILVA website redesign featuring DSMZ Digital Diversity branding.

As part of the integration with DSMZ Digital Diversity, we redesigned SILVA’s website to match the design and branding of other databases within the D3 (Fig. 2). This ensures a consistent user experience across all D3 databases while maintaining SILVA’s distinct identity. The new interface adopts standardized navigation patterns, color schemes, and layout principles that users will recognize from other D3 resources, facilitating easier transition between databases. We plan to implement further improvements to navigation and user experience, as well as enhanced interoperability between SILVA and other resources within the D3 (see Future Directions).

### Addition of classifiers

To better serve the research community, we implemented a new feature on our website—the generation of customized QIIME2 [35], Kraken2 [36], and DADA2 [37] formatted classifiers based on the latest SILVA taxonomy and reference datasets. This includes the possibility to generate region- and habitat-specific classifiers via the TestPrime tool (QIIME2; closed beta at time of writing) or to download ready-to-use classifiers directly from the SILVA Archive (QIIME2, DADA2, and Kraken2).

Typically, researchers are encouraged to either download classifiers from public repositories or generate and adapt them to their own needs. The first option may result in classifiers that are too generic and not well adapted to the individual project. The second option requires technical expertise and knowledge of the proper use of the corresponding tools, which can be a barrier for many users. Therefore, the integration of classifiers into the SILVA pipeline provides multiple benefits for the users, including (i) access to up-to-date classifiers that are directly linked to the latest SILVA release; (ii) availability of region- and habitat-specific classifiers, e.g. tailored to individual primer pairs or environmental contexts; (iii) significant time and computational resource savings by enabling classifier generation on high-performance computing platforms provided by SILVA; (iv) seamless integration with *in silico* primer design via the TestPrime tool, ensuring that classifiers are optimally tailored to experimental designs; and (v) reduction of barriers for beginners yet customizable for advanced users.

To generate QIIME2-based classifiers, we used the RESCRIPt [38] and Clawback [39] plugins, as well as the Ready-to-wear repository [39]. This enables optimization of classifiers in two ways: region-specific optimization—adaptation of the reference database to amplicon regions, ensuring robust and accurate taxonomic classification [40, 41] and habitat-specific optimization—weighting classifiers according to expected community compositions in particular environments [39].

For DADA2, we used the native implementation of the naïve Bayesian classifier method [42]. This allows the creation of training sets based on the SILVA datasets, optionally including species-level information. While species-level annotations can improve classification depth, the SILVA database does not classify to the species level due to SSU and LSU resolution limits, requiring careful interpretation of results. As with the QIIME2 classifiers, our archive provides training sets for DADA2, ensuring that users can start directly with reliable data without the need for extensive preprocessing.

To generate the SILVA-based database for Kraken2 classification, we followed the SILVA installation script and converted the taxonomy into the Kraken2 format [36]. To refine taxonomic assignments, we also provide databases generated with Bracken, using the same read length settings as in the Kraken2 repository [43]. This ensures comparability with existing workflows while providing the advantage of up-to-date and SILVA-specific data.

### FAIR data and open source strategy

As an ELIXIR Core Data Resource and Global Core Biodata Resource, SILVA is dedicated to promoting open science and FAIR data. All data provided by SILVA is published under the Creative Commons Attribution 4.0 (CC-BY 4.0) license. To further advance data reuse, DOIs have been implemented for each SILVA release. Addition of DOIs to all associated files within a SILVA release allows researchers to cite the specific dataset that have been used in their analysis, as well as enabling developers who integrate SILVA data to build their tools to provide proper attribution. Each DOI provides rich metadata covering the release version, subunit, dataset (Parc, Ref NR, or Ref NR 99) and a short content description (e.g. fasta or metadata). This allows users to easily identify the origin of SILVA data. DOIs are provided for all 138.X releases and will be included in all upcoming releases. For historic SILVA releases, DOIs will be added gradually.

To support the open source software development and foster the reuse of code, SILVA developed an open-source strategy. Current software developments are designed to support a potential reuse by the scientific community, taking into account an open license, freedom from legal constraints and accurate documentation. As an example, the recently developed TaxMap application has been published as open source (AGPLv3; https://code.dsmz.de/eukmap/). All newly developed web components will follow this example. Due to legal constraints, developments that utilize old code cannot be shared until these have been completely redeveloped.

### TaxMap

**Figure 3.**
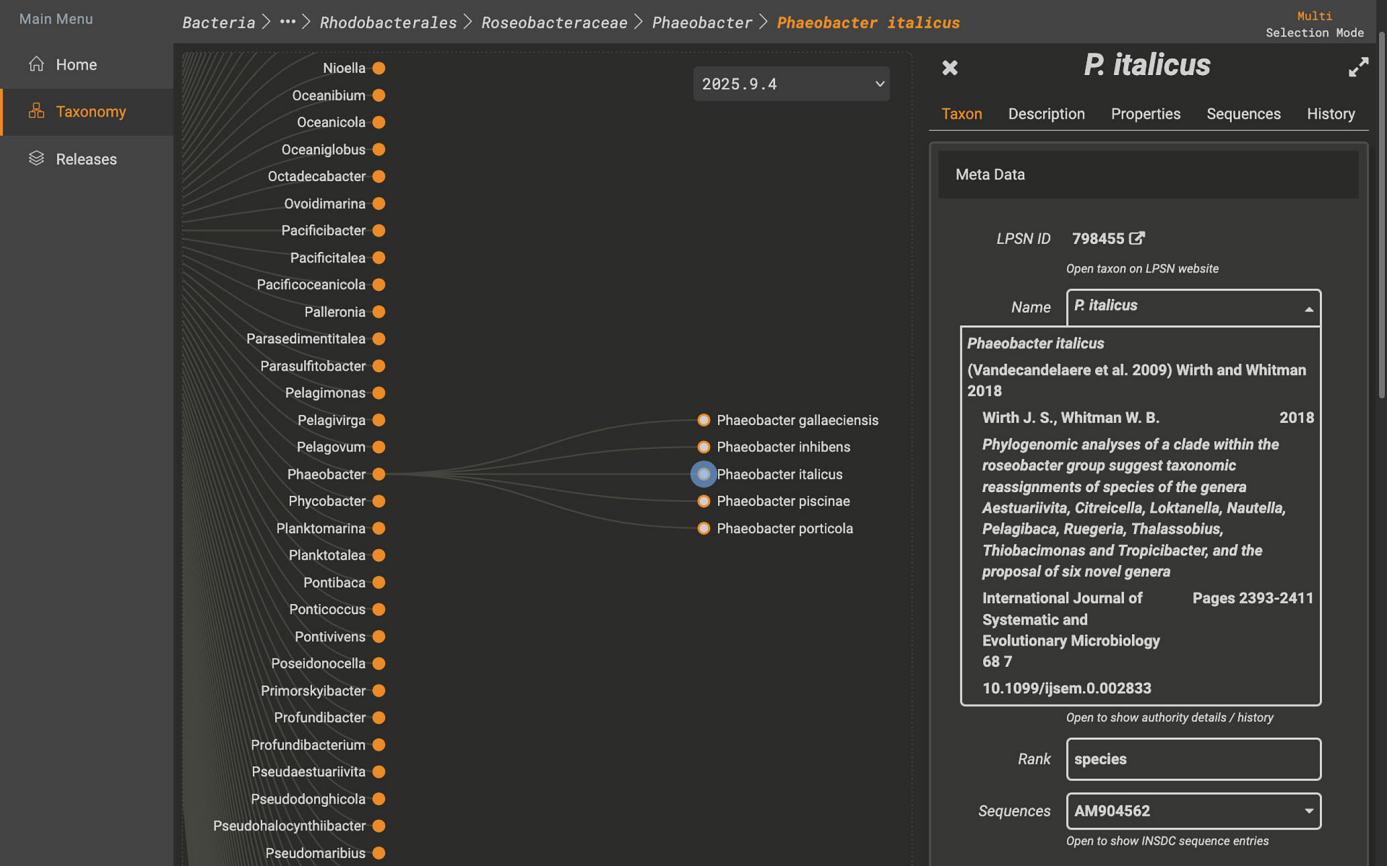
TaxMap website interface reflecting LPSN taxonomic hierarchy, showing taxon details for *Phaeobacter italicus*.

The EukMap application [4] was originally developed by the SILVA team as part of the UniEuk project (https://unieuk.net) for their eukaryotic taxonomic framework. This application was adopted to provide a visual version of LPSN’s hierarchical classification, facilitating curation of bacterial and archaeal taxa in SILVA trees. The names in TaxMap are based on validly published names according to the rules of ICNP, correct names according to LPSN, as well as synonyms. The adapted version is named TaxMap and is available via https://taxmap.dsmz.de. TaxMap facilitates the navigation of the prokaryotic taxonomy derived from LPSN’s data collection. A selection of the most important LPSN metadata is available in the application together with a link to further details on the corresponding taxon page on the LPSN website (Fig. 3). It is important to note that TaxMap does not continuously fetch LPSN data; however, it does contain periodic snapshots and provides them as releases. The preservation of releases will serve to establish an archive of historical taxonomic opinions. This will also improve the reproducibility of SILVA by reflecting LPSN names used in the curation of each specific release. The selection of taxa in TaxMap is derived from the data available via the LPSN API. Taxa with missing descendants are not imported as a consequence of technical constraints. The application requires a tree-based taxonomy.

### Future directions

Here, we present a major direction outlining key areas and ideas for the future development of SILVA as a leading rRNA-based classification resource in microbiology. As one of the top priorities, we plan to improve the classification workflow by integrating improved approaches for large-scale phylogenies based on SSU and LSU genes, ensuring more accurate taxonomic assignments across the three domains of life. To maintain the most up-to-date and reliable taxonomies, SILVA will continue reconciling 16S- and genome-based prokaryotic taxonomies by adopting GTDB taxonomic assignments and nomenclatural information from LPSN. Furthermore, we will continue to improve the alignment between SSU and LSU classifications by establishing representatives in both trees and reconciling their taxonomies, particularly for eukaryotes (e.g. fungi).

To further align 16S- and genome-based classifications from genus to phylum, redefined taxonomic boundaries based on 16S rRNA gene identity [44] will be investigated for incorporation into future SILVA releases. The newly proposed boundaries overlap across taxonomic ranks and correlate with relative evolutionary divergence (RED), a metric used in the GTDB taxonomy for classifying taxa from genus to phylum [2]. Additionally, a recent study demonstrated the limitations of 16S rRNA as a species-specific marker due to insufficient diversification within genera in some lineages, resulting from evolutionary stasis caused by horizontal gene transfer [45]. This further supports that 16S rRNA is more reliable at the genus level and reinforces why SILVA provides classifications only down to genus.

Another top priority is the scalability challenges across all workflow stages due to the rapidly growing volume of data. SILVA is committed to meet this need by modernizing its release pipeline. This will enable more efficient, reproducible, and scalable processing of data and allow reliable annual data releases to serve the scientific community best. To improve interoperability and facilitate integration with other resources, we plan to implement a knowledge graph built upon the DSMZ Digital Diversity Ontology (https://bioportal.bioontology.org/ontologies/D3O). This approach will build on existing implementations for Bac*Dive*, BRENDA and Media*Dive* and help to interlink taxonomic classifications, sequence data, and associated metadata, via a SPARQL endpoint and make SILVA data machine-interpretable, e.g. for Retrieval-Augmented Generation (RAG). These developments will improve SILVA integration with other resources including D3 and support reuse across different platforms.

Future plans also include developing of a taxon history tool to track taxonomic changes between releases, expanding functionality of the TaxMap, integrating sequence retrieval based on isolation sources using DSMZ-developed ontology, adding sequence cluster content information to the website, and expanding metadata and external links from prominent taxonomic and nomenclatural resources. We are committed to ensure that our upcoming changes address the demands of the research community and reflect ongoing developments in taxonomic research.

## Supplementary Material

gkaf1247_Supplemental_File

## Data Availability

SILVA releases are available via https://www.arb-silva.de.
