# Supplementary material for: SILVA in 2026: a global core biodata resource for rRNA within the DSMZ digital diversity
Source: Nucleic Acids Res. 2025 Nov 18;54(D1):D334–41. doi: 10.1093/nar/gkaf1247 (PMC12807666; doi:10.1093/nar/gkaf1247)
Supplement: gkaf1247_Supplemental_File [file gkaf1247_supplemental_file.pdf]

SILVA in 2026: A Global Core Biodata Resource for rRNA within the DSMZ Digital Diversity

Maria Chuvochina[1], Jan Gerken[1], Martinique Frentrup[1], Yeliz Sandikci[1], Robin Goldmann[1], Heike M. Freese[1], Markus Göker[1], Johannes Sikorski[1], Pablo Yarza[4], Christian Quast[5], Jörg Peplies[5], Frank Oliver Glöckner[2,3] and Lorenz Christian Reimer[1]

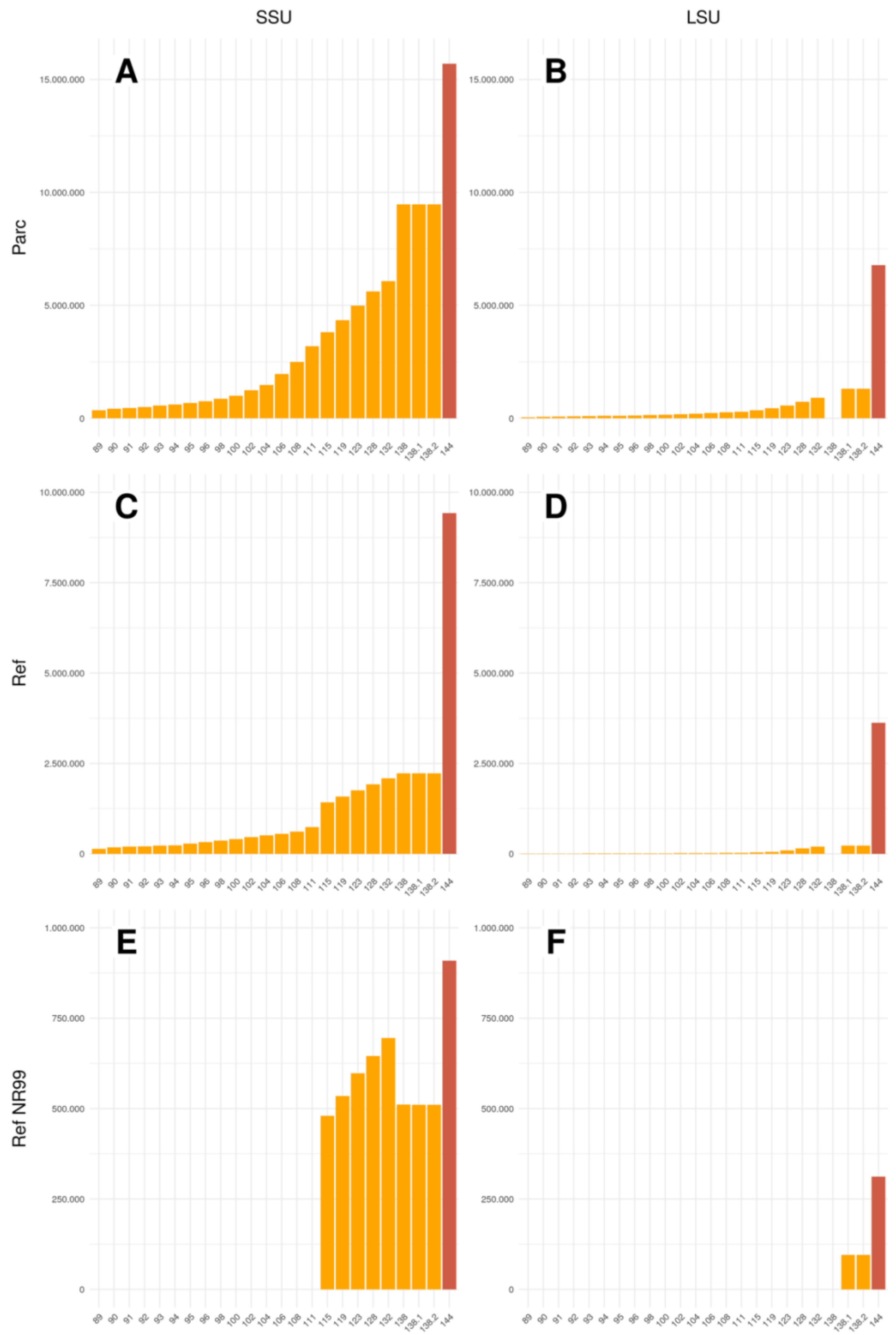

Supplementary Figure 1. SILVA database growth across different releases, dataset subsets and rRNA subunits. (a) Parc SSU, (b) Parc LSU, (c) Ref SSU, (d) Ref LSU, (e) RefNR99 SSU, and (f) RefNR99 LSU. Parc contains all sequences available in SILVA, Ref is a curated subset of high-quality, non-redundant sequences, and RefNR99 is clustered at 99% sequence identity. SSU refers to small subunit rRNA genes (16S/18S), while LSU refers to large subunit rRNA genes (23S/28S). The numbers shown for the release 144 are provisional (before manual curation).
